# Supplementary material for: Prevalence and mechanisms of high-level carbapenem antibiotic tolerance in clinical isolates of Klebsiella pneumoniae
Source: PLoS Pathog. 2026 Jan 30;22(1):e1013919. doi: 10.1371/journal.ppat.1013919 (PMC12880740; doi:10.1371/journal.ppat.1013919)
Supplement: S1 Text — Fig A. Tolerance screen validation with benchmark isolates. Strains were exposed to meropenem (10 µg/mL) as described in methods. At the indicated time-points, 10 µL of the cell suspension was spotted on BHI agar and supplemented with KPC, followed by 24 hours of incubation at 37 ˚C. Fig B. Complementation of the ∆mltB phenotype. The indicated strains were grown overnight in BHI, then diluted 10-fold into fresh BHI containing meropenem (10 µg/mL) and 40 ng/mL anhydrotetracycline. Viability was determined by serial dilution and spot-plating after 24 hours of incubation. Shown are raw data points for 5 biological replicates for each strain. Statistical significance was determined by Mann Whitney test (*p < 0.05). Fig C. Tolerance phenotypes of CESR mutants. Time-dependent killing experiments (10 µg/mL meropenem) were conducted in BHI medium. Statistical analysis (Kruskal-Wallace test, p < 0.001) revealed statistically significant differences in viability (12 hour time point) for all mutants vs. WT; ∆4 vs. any triple mutant was not statistically significant. Fig D. Complementation of the ∆CESR phenotypes. The indicated strains were grown overnight in BHI, then diluted 10-fold into fresh BHI containing meropenem (10 µg/mL) and 40 ng/mL anhydrotetracycline and incubated for 24 hours. Viability was determined by serial dilution and spot-plating. Shown are boxplot and raw data points for at least 5 biological replicates for each strain. Fig E. Meropenem induces the Rcs phosphorelay. A) The PrprA-lacZ reporter strain was spread on plates containing the chromogenic LacZ substrate x-gal. A filter disk was placed in the middle containing either meropenem or kanamycin. Note blue halo around the meropenem disk, in the sub-MIC area beyond the zone of inhibition. B) RcsB was overexpressed from an arabinose-inducible promoter in the PrprA-lacZ background, followed by plating on LB with X-gal. (DOCX) [file ppat.1013919.s001.docx]

**Supplementary Figures**

**
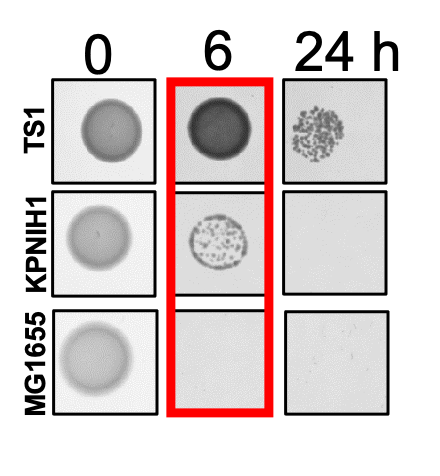
**

**Fig A. Tolerance screen validation with benchmark isolates.** Strains were exposed to meropenem (10 µg/mL) as described in methods. At the indicated time-points, 10 µL of the cell suspension was spotted on BHI agar and supplemented with KPC, followed by 24 hours of incubation at 37 ˚C.

**Fig B. Complementation of the ∆*mltB* phenotype.** The indicated strains were grown overnight in BHI, then diluted 10-fold into fresh BHI containing meropenem (10 µg/mL) and 40 ng/mL anhydrotetracycline. Viability was determined by serial dilution and spot-plating after 24 hours of incubation. Shown are raw data points for 5 biological replicates for each strain. Statistical significance was determined by Mann Whitney test (*p < 0.05)

**
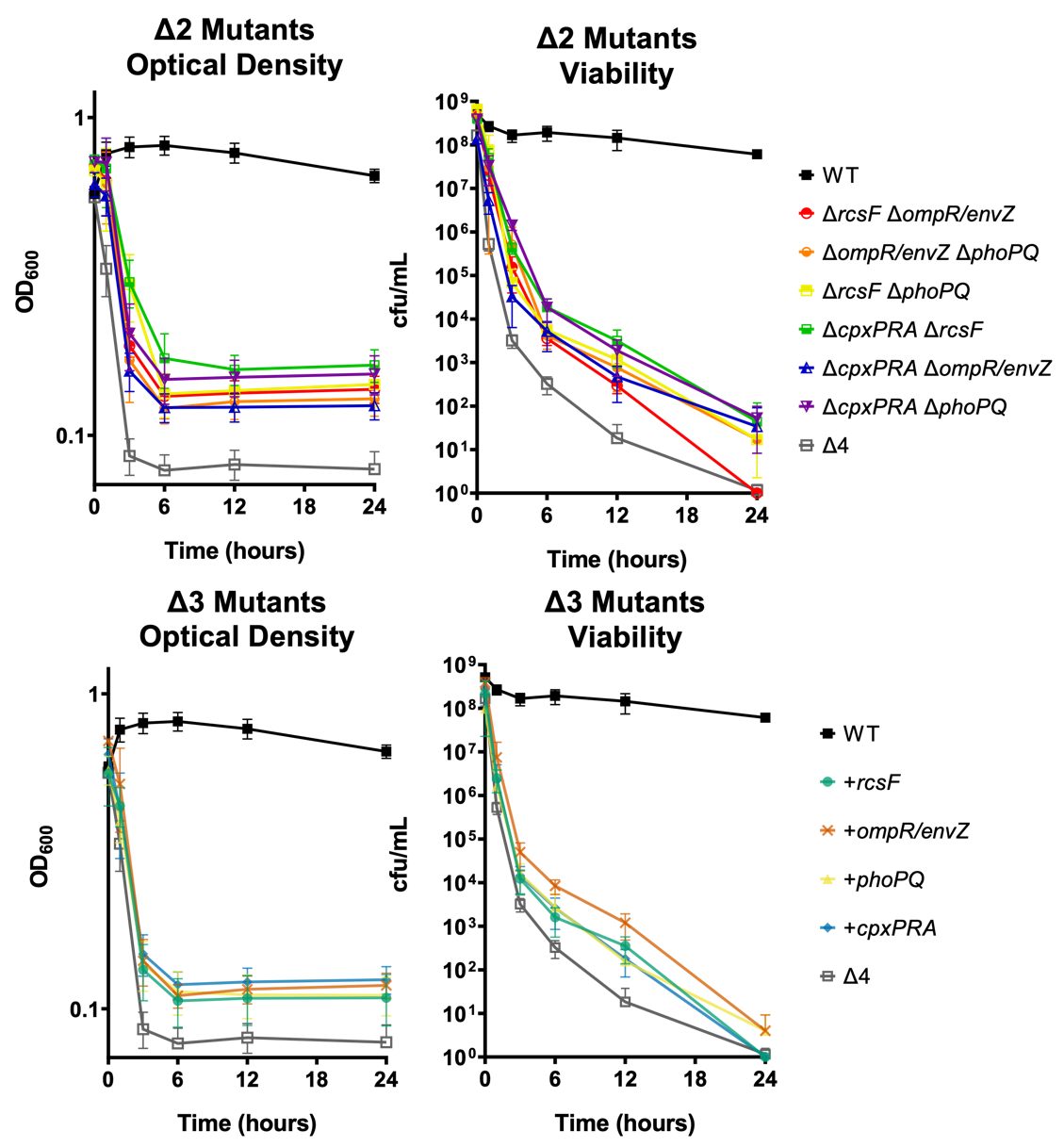
**

**Fig C. Tolerance phenotypes of CESR mutants.** Time-dependent killing experiments (10 µg/mL meropenem) were conducted in BHI medium. Statistical analysis (Kruskal-Wallace test, p < 0.001) revealed statistically significant differences in viability (12 hour time point) for all mutants vs. WT; ∆4 vs. any triple mutant was not statistically significant.

**Fig D. Complementation of the ∆CESR phenotypes.** The indicated strains were grown overnight in BHI, then diluted 10-fold into fresh BHI containing meropenem (10 µg/mL) and 40 ng/mL anhydrotetracycline and incubated for 24 hours. Viability was determined by serial dilution and spot-plating. Shown are boxplot and raw data points for at least 5 biological replicates for each strain.

**
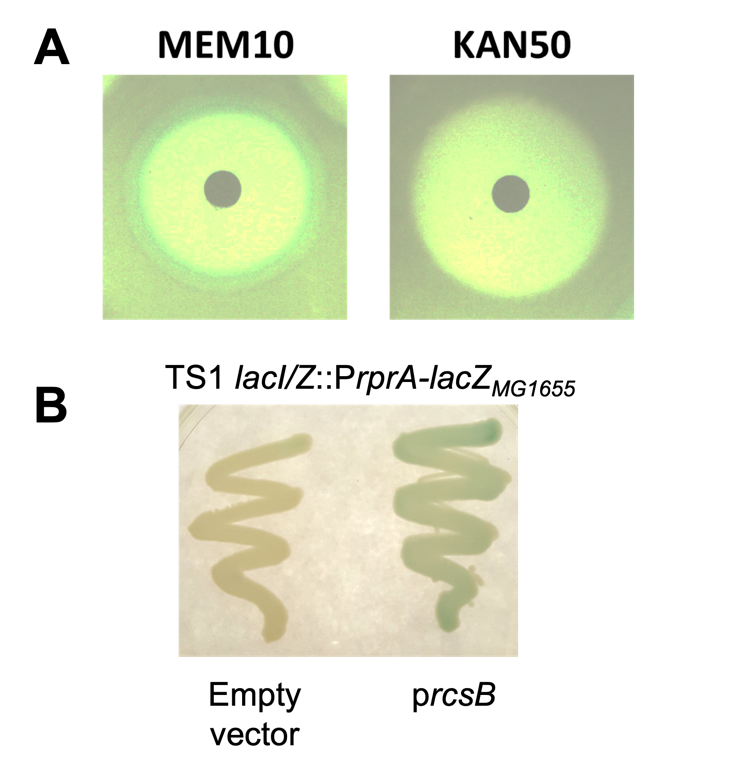
**

**Fig E. Meropenem induces the Rcs phosphorelay. A)** The P_rprA_-*lacZ* reporter strain was spread on plates containing the chromogenic LacZ substrate x-gal. A filter disk was placed in the middle containing either meropenem or kanamycin. Note blue halo around the meropenem disk, in the sub-MIC area beyond the zone of inhibition. B) RcsB was overexpressed from an arabinose-inducible promoter in the P_rprA_-*lacZ* background, followed by plating on LB with X-gal.
