# Supplementary material for: Prevalence and mechanisms of high-level carbapenem antibiotic tolerance in clinical isolates of Klebsiella pneumoniae
Source: PLoS Pathog. 2026 Jan 30;22(1):e1013919. doi: 10.1371/journal.ppat.1013919 (PMC12880740; doi:10.1371/journal.ppat.1013919)
Supplement: Attachment — Submitted filename: Reviewer_Response_KlebTol2.docx [file ppat.1013919.s008.docx]

**Editor Assessment:**

Thank you for submitting your manuscript to PLOS Pathogens. After careful consideration, we feel that it has merit but does not fully meet PLOS Pathogens's publication criteria as it currently stands. Therefore, we invite you to submit a revised version of the manuscript that addresses the points raised during the review process.

We thank the editor for their assessment and have responded to the reviewer comments point by point below.

Of particular note, the fluctuation test needs clarification to improve the understanding of this technique.

We have supplied additional explanations and expanded the methods section for the fluctuation test.

Addressing the novelty of these findings, there are previous reports not mentioned in the manuscript that have implicated the factors identified here are important for cell envelope stress.

We have added some more information to the discussion section outlining the novelty of the findings.

Furthermore, as the current focus of tolerance is on growth rate, the results presented here showing that stress response mechanisms preserve non-growing cells should be explained in the context of growth rate to show how these findings change our current understanding of tolerance.

The role of growth rate in tolerance is a somewhat complex issue. Indeed, tolerance can result from slow/non-growth, but this is not always the case. In our system (carbapenem tolerance in Enterobacterales afforded by retaining structural integrity of the outer membrane), survival is somewhat uncoupled from growth rate, i.e. spheroplasts are metabolically active, keep growing (expanding) but do not divide and apparently survive by constantly repairing damage induced by the antibiotic. We have added additional language in the introduction to dissect these different strategies, by inserting the sentence *“Unlike in the case of heterotolerance (persistence), spheroplast survival is afforded not by preventing antibiotic-induced damage through dormancy or reduction in growth rate, but rather by damage management through activation of stress responses”* with proper references.

**Part I - Summary**
Please use this section to discuss strengths/weaknesses of study, novelty/significance, general execution and scholarship.

**Reviewer #1:** Cross et al. examines the prevalence of carbapenem tolerance in a large panel of Enterobacterales bloodstream isolates. They show that Klebsiella pneumoniae strains exhibited the highest overall tolerance to the last line antibiotic meropenem. The authors employed an existing transposon library and elegant microscopy to uncover the mechanism of meropenem tolerance in K. pneumoniae. Their study links tolerance to envelope-stress systems (Rcs, PhoPQ, Cpx, OmpR/EnvZ) and identifies lytic transglycosylases as anti-tolerance factors, connecting survival to spheroplast formation and recovery. The mechanistic studies were well controlled with appropriate complemented strains in the supplementary materials.
A major strength is the high-throughput tolerance screen that still works despite MIC and growth-rate differences typically observed in panels of clinical isolates. Using a single fixed meropenem concentration (appropriate for time-dependent β-lactam killing) and quenching with a carbapenemase to avoid wash steps are innovative design choices. The screen’s rankings translated well to quantitative time-kill assays and will be of particular interest to the antibiotic tolerance community.
The manuscript would benefit from some additional discussion on if spheroplasts could contribute to carbapenem treatment failure in patients. Overall, this is an important study that shifts the focus beyond antibiotic resistance and highlights the need for understanding and targeting tolerance mechanisms.

We thank the reviewer for their thoughtful and positive perspective.

**Reviewer #2:** In their manuscript entitled “Prevalence and mechanisms of high-level carbapenem antibiotic tolerance in clinical isolates of Klebsiella pneumoniae”, the authors characterize meropenem tolerance in clinical isolates in a range of species. Finding K. pneumoniae to be particularly tolerant to meropenem, subsequent work was focused on characterizing the genetic requirements for meropenem tolerance in K. pneumoniae. The work presented here provides molecular insight into the signaling pathways promoting tolerance in the presence of meropenem and potentially other beta-lactam antibiotics. I really appreciated the medium throughput assay for tolerance phenotypes in their collection of clinical K. pneumoniae isolates. The use of the carbapenemase to scavenge meropenem for the plating assay was very clever.

We thank the reviewer for their positive assessment!

Altogether, the results suggest a collective and potentially sequentially activated stress response that is protective to K. pneumoniae grown in the presence of meropenem. At the same time, why and how this response leads to a halt in cell growth remains an open question, but an important one given the established connection between tolerance and slow growth at the population and single cell level. Is it possible that the cell envelope stress response preserves viability just long enough for cells to enter a slow growing, tolerant state? Finally, the rationale for some of the experiments, particularly the unusual use of the fluctuation test, is unclear and there are additional instances where more precise use of language and/or more clarity would be welcome.

The reviewer raises an important point about growth rate. Indeed, tolerance is very often correlated with growth rate. However, in our system (spheroplast-mediated carbapenem tolerance), it is more an inoculum effect, where the growth yield rather than growth rate determines viability: Spheroplasts expand at the same rates as untreated cells, see also PMID: 31285232 and OD measurements in the current manuscript, and survival is ensured by reaching carrying capacity. We have included additional discussion of growth rate vs. tolerance, and added clarity to the fluctuation test in the revised manuscript.

**Reviewer #3:** The study by Cross et al. explores the prevalence and mechanisms behind carbapenem tolerance among clinical isolates of Klebsiella pneumoniae. Through screening of 271 bloodstream isolates Cross et all. identify higher prevalence of tolerance in K. pneumoniae isolates than in E. coli. A hyper-tolerant strain, TS1, survived nearly all meropenem exposure by forming cell wall‑deficient spheroplasts that recovered once the drug was removed. Using transposon sequencing, the authors identified cell envelope stress response systems (PhoPQ, Cpx, Rcs, OmpR/EnvZ) as essential for spheroplast stability and recovery, while the lytic transglycosylase MltB disrupted tolerance.

The area under scrutiny is clinically relevant and timely. Our understanding of the breadth of tolerance in clinically relevant context is important and the possibility that targeting stress response pathways or enzymes like MltB could aid eradication of tolerant infections is of broad interest.

We thank the reviewer for their enthusiasm for our work.

**Part II – Major Issues: Key Experiments Required for Acceptance**
Please use this section to detail the key new experiments or modifications of existing experiments that should be absolutely required to validate study conclusions.

Generally, there should be no more than 3 such required experiments or major modifications for a "Major Revision" recommendation. If more than 3 experiments are necessary to validate the study conclusions, then you are encouraged to recommend "Reject".

**Reviewer #1:** NA

**Reviewer #2:** 1. The fluctuation test is an unusual way to examine what appear to be non-heritable phenomena. Is the idea to look at variation in single cell responses? Overall, this section would be improved by revising for clarity as well as improving the presentation of the data. Specifically:
a. While there are details of the experimental method in the results sections, I didn’t see a methods section for this experiment. That should be included for reproducibility. How many wells were examined for each strain? Based on the graphs provided it looks like there are more replicates of the Δ4 in the 1 cell condition than WT.

We apologize for the oversight and have now added a description of the methods. Indeed, there are different numbers of replicates, since generating wells that start with 1 cell is stochastic: we dilute our starting culture to homeopathic levels, which on average yields 1 cell in each well that starts growing. Naturally this means that every experiment differs in the number of wells were growth is observed.

b. Is OD600 a truly robust or meaningful readout for spheroplast populations when the Δ4 population doesn’t appear to readily generate spheroplasts (Fig 5C)?

Yes, this is still meaningful. WT tolerance exclusively relies on spheroplast formation, so OD600 readings are an accurate proxy. The ∆4 does not readily generate spheroplasts, but the degree of lysis can still be quantified via OD600.

c. There’s only one value on the Y-axis. Perhaps a supplemental figure could be included that show more clearly the areas between the meropenem added and when CV was calculated?
We have changed the axis to include the entire OD600 spectrum we measured.

2. The authors need to address how their findings fit into the current understanding of factors underlying antibiotic tolerance, particularly with regard to growth rate. E.g. is expression of the stress response systems protective immediately following challenge with meropenem?

The type of tolerance we study here is a little different from canonical persistence – spheroplasts are fully metabolically active (PMID: 41282133) and keep expanding upon antibiotic exposure (PMID: 31285232, PMID: 25884840). The cell envelope stress responses do not shut down growth, but likely rather constantly repair damage induced by the antibiotic. This is physiologically very different from persistence, and we thus only included only a short discussion of these other manifestations of tolerance.

**Reviewer #3:** Tolerance assay:
It appears that only a single replicate is shown. Given that the rest of the manuscript relies on these observations, at least 3 biological replicates should be carried out and presented.

We have actually already done what the reviewer asked for– our tolerance assessment was based on 3 independent replicates of the entire screen (this is shown in Table S1 where the assessment for all 3 replicates is given (columns G-L). We included a visual representation in Fig. 1B just as an example of what the assay looks like.

Fig 2B and 4C:
Please provide the number of cell frames imaged and information on biological replicates. Additional panels from the sample showing the prevalence of the observed phenotypes should be included in supplementary.

We have added this information to the manuscript.

Statistical analysis for several figures is missing and should be included:
- Ln 120: statistical significance of the correlation is mentioned but not shown. I agree with the conclusion that biological significance is not present however given the analysis is mentioned, it should be included in the legend along with its results.

This information is already in the manuscript (we report spearman co-efficient with p value), but was a little hidden at the end of the sentence. We have now added it more directly to the statement about statistical significance.

- Fig 6B: statistical analysis is missing, please correct.

Statistical analysis has been added to Figure 6B.

- Fig 5: Please add details on replicates and statistics.

We have added this information to the capture of Figure 5.

**Part III – Minor Issues: Editorial and Data Presentation Modifications**
Please use this section for editorial suggestions as well as relatively minor modifications of existing data that would enhance clarity.

**Reviewer #1:** 1. Presumably spheroplasts are very susceptible to other stresses. Can the authors comment on if these spheroplast could be a mechanism of tolerance that occurs during treatment of bloodstream infections.

This is a question we are very much interested in. Our previous study revealed that spheroplasts do form in human serum (PMID: 31285232 ), but this was heat-inactivated serum, i.e. without active complement. We currently do not know whether spheroplasts are more or less resistant to e.g. antimicrobial peptides or complement, but are working on a study addressing this issue (in the context of a larger study on the role of stress responses in infection outcomes).

2. Related: Are the tolerant K. pneumoniae spheroplasts still capsulated? If so, could the capsule provide protection from killing by whole blood?

This is also a very interesting comment. We do not know if spheroplasts are still capsulated, but assume they produce at least colanic acid, since Rcs is induced by meropenem.

3. Line 413-415: Interpretation of the fluctuation test results should be expanded to improve clarity. Does this result suggest that meropenem tolerant cells (spheroplasts) are pre-existing in this population before the addition of the drug? If so, could similar experiments be performed in more physiologically relevant conditions (serum or blood) to see if the trend holds up?

Indeed, the interpretation is that tolerant cells (not quite spheroplasts, but cells with enhanced propensity to form spheroplasts) are pre-existing in the population. We have added a more thorough discussion of how to interpret the fluctuation test data, both in the Results and in the Methods section. Doing these experiments in serum or blood presents technical challenges (OD measurements are not possible), and would make these data less comparable to the other experiments, which were performed in BHI. That said, we would argue that BHI is a physiologically relevant, “host-like” condition as well.

**Reviewer #2:** Major Comments:
1. The authors present TN-seq and mutant data to support a requirement for cell envelope stress response systems in meropenem tolerance. This work was performed in the TS1 strain, which exhibited higher tolerance to meropenem than E. coli MG1655 or another K. pneumoniae strain, KPNIH1. These cell envelope stress systems are broadly conserved in Gram-negative bacteria and not unique to TS1. How do these systems contribute to the underlying base-line differences in tolerance between organisms/strains? Do the author’s have any insights into why TS1 is more tolerant than other strains?

We wish we did! This is a very intriguing question that is unfortunately beyond the scope of this study. The fact that different strains representing the same species have sometimes dramatically different tolerance level will lend itself to comparative genomics in a future study.

2. Is Meropenem special? The title suggests this phenomenon holds for all carbapenems but I don’t think this has been tested. If Meropenem is special or is the only drug tested the title should be revised to reflect this.
Meropenem is essentially a model carbapenem for our purposes (and also the most widely used carbapenem in clinical therapy).

3. Do the authors have any thoughts as to why overexpression of rcsB seems to impair survivability in a WT background? (Fig 6 B).

Like the reviewer we were intrigued by this observation, but at this point we do not have a mechanistic explanation. Cell envelope stress response induction often carries with it fitness trade-offs (this is one of the reasons why most of the are induced by stresses rather than constitutive), and we suspect that expressing RcsB at supra-physiological levels represents such a trade-off.

4. The author’s find that loss of MltB and SltY, both of which should lead to accumulation of glycan strands, increase tolerance. I believe this is not unexpected result based on Cho et al 2014, who found that defects in the E. coli soluble lytic transglycosylase, SltA are protective in the presence of mecillinam, in accordance with their futile cycle model for beta-lactam killing in E. coli. This same paper indicates that a defect in mltB “imparts a fitness advantage in the presence of meropenem” which I believe similar to this study’s finding in K. pneumoniae if one assumes tolerance and resistance share overlapping mechanisms. Regardless, the relationship between this finding and those of Cho et al should be addressed in the results and/or discussion.

The reviewer brings up an interesting point. The big difference is that in the Cho et al system, an *slt* mutation in *E. coli* increases resistance, but only when paired with FtsZ overexpression. In contrast, an *slt* mutant in a wt background is actually hypersensitive to mecillinam. So the physiological setup is somewhat different from ours. Their single slt mutant actually confers hypersensitivity to an antibiotic targeting a specific PBP. This is in contrast to Klebsiella (our data), where an *mltB* mutant confers high tolerance (and actually slightly increased resistance). Following the reviewer’s recommendation, we have now expanded our discussion of futile cycling in the context of LTG activity and our phenotypes as well.

Minor Comments
1. Line 25: “novel” might not be the correct word. Most of these genes have been previously implicated in the response to cell envelope stress. Could just say identified cell envelope stress related factors as important for tolerance.

This point is well taken, we have deleted the word “novel”

2. Line 40. Clarify that tolerance means “survive but do not grow”

Following the reviewer’s recommendation, we have rephrased to “represents a state where a large fraction of susceptible bacteria survives in the presence of an antibiotic for extended periods, but does not proliferate”

**Reviewer #3:** Data/Analysis:
- Fig 3B: was a cut-off implemented for prioritizing genes for further validation?

Yes, we focused on the top 10 % of TnSeq hits.

- Ln 230-232: Given the validation hits seem to have been selected by picking hits that have exhibited low insertion rate after exposure – how were the two ‘survival supporting’ hits picked up? Surely those would have shown a complete opposite phenotype on the insertion rates? … I could find neither MltB or OsmB in table S4 so this cannot be extrapolated from there.

We apologize for this oversight – the annotation in the table is the one taken from the original library, which lacks annotation for some genes. We have fixed this.

- Fig 4B: TS1 appears to afford almost the same survival as KPNIH1 mltB mutant - is the TS1 deficient or lacking the expression the mltB or is this phenotype arising from another variation?

This is a very interesting question. TS1 does appear to have a fully functional MltB, so this is not the reason for its high tolerance. Future work will reveal potential strain-specific differences in expression/activity of these enzymes that will provide insight into their role during sacculus degradation.

- It is unclear why the stress responses are studied in TS1 but the prior section is done in KPNIH1, especially since the KPNIH library seems to exist – why were different mutants created in the TS1 background and the KPNIH library not exploited?

This has historical reasons. We chose the TS1 mutant for the initial screen due to its high tolerance. We then used the KPNIH mutant for a quick validation of a large number of hits (since the disruption mutants are immediately available in the ordered transposon library). However, since we are ultimately interested in the hypertolerant TS1 isolate, we constructed and tested defined mutants in our most prominent hits (the CESRs) in this isolate as well.

Figure/Table comments:
- Supplementary Table 1: Please clarify what this table is showing. Given Ln 143, this table appears to link to tolerance phenotypes, however its first mention at Ln65 which discusses resistance makes the data presented in this table difficult to place.

We agree with the reviewer that this was a bit confusing. This table shows the summary statistics for our tolerance categorization. We have made this more clear in the table caption. We have also re-arranged the table numbering to make more sense (the full panel information is now shown in Table S1, summary statistics in S2).

- Ln 163 and Table S2: MIC values are mismatched for TS1, please correct.

We apologize for this oversight – these are both correct as we found a 2-fold variation in our several replicates of the MIC assay (which is not unusual for MIC assays). We now report the range as “0.0625 – 0.1 µg/mL”.

- Fig 3C: The y-axis is a little misleading. Are those fractions of survival of CFU per mL?

Yes, this is survival fraction (CFU/mL after exposure divided by CFU/mL before exposure). We have now clarified this in the legend.

- Ln 400 and Fig 7: legend do not match. The figure implies that CV calculations were done at the blue dotted line timepoint while the main text suggests this was carried out at the red line timepoint. Please clarify.
This was a typo, which we have corrected!
Textual comments:
- Ln 64–65: Please reference for sentence starting “In some bacteria…” – done!
- Ln 146: Please remove the stray “.” after “species”. – corrected!
- Ln 244: Please define LTG. – done!
- Ln 309: Please remove the aberrant “mutant” word. – corrected!
- Ln 318: Please define CESR. – done!

- Fig S4: appears in the text prior to FigS3, please correct the order.

Done!

- Ln 318-319 : ‘CESR single deletion mutants formed spheroplasts, albeit at much lower numbers compared to WT.’ is followed by a statement saying that 2/4 are exceptions. I would consider removing the above highlighted sentence and covering each deletion separately, especially given that half of the 4 mentioned are actually not what is described, essentially implying there is no single unique trend (beyond the overall tolerance effect)
- Ln 319-325: The structure of this paragraph is a little disjointed and hard to follow. I would suggest discussing all points on the rcs system first and then moving all the ompR/envZ.

We agree with both of the reviewer’s suggestions and have reworded this passage accordingly.

- Ln 385: please remove ‘both’ when discussing 3 species.

Corrected!

- Throughout the manuscript hyphenation is often missing between ‘number(-)fold’.

Corrected!
