# Supplementary material for: Prevalence and mechanisms of high-level carbapenem antibiotic tolerance in clinical isolates of Klebsiella pneumoniae
Source: PLoS Pathog. 2026 Jan 30;22(1):e1013919. doi: 10.1371/journal.ppat.1013919 (PMC12880740; doi:10.1371/journal.ppat.1013919)
Supplement: Attachment — Submitted filename: Reviewer_response_final.docx [file ppat.1013919.s009.docx]

Reviewer #2: While I understand that meropenem is a representative carbapenem, the data do not support the idea that K. pneumoniae will respond to other carbapenems in the same way. To avoid confusion, I respectfully request title be revised to either specify that drug tested was meropenem or at least to indicate that only one carbapenem was tested. E.g. "Prevalence and mechanisms of high-level antibiotic tolerance to a representative carbapenem in clinical isolates of Klebsiella pneumoniae"

We appreciate the reviewers concern, but it is actually accurate as written (we do not claim that this covers all carbapenems; the numberless phrase “carbapenem antibiotic tolerance” is semantically accurate even if only one compound is considered). While this may seem like a minor point, the flow of the title just does not match what we have here if we try to squeeze in more information.

Reviewer #3: There is a lack of italicization on ln 40 for Klebsiella pneumoniae

Fixed!
